# Functional Differentiation of the Duplicated Gene *BrrCIPK9* in Turnip (*Brassica rapa* var. *rapa*)

**DOI:** 10.3390/genes15040405

**Published:** 2024-03-26

**Authors:** Haotong Kang, Yunqiang Yang, Ying Meng

**Affiliations:** 1 Key Laboratory of Plant Resources Conservation and Utilization, College of Biological Resources and Environmental Sciences, Jishou University, Jishou 416000, China; kanght228@163.com; 2 The Germplasm Bank of Wild Species, Kunming Institute of Botany, Chinese Academy of Sciences, Kunming 650201, China; yangyunqiang@xtbg.ac.cn; 3 Institute of Tibetan Plateau Research at Kunming, Kunming Institute of Botany, Chinese Academy of Sciences, Kunming 650201, China

**Keywords:** turnip, duplicated gene, NAF, abiotic stress, functional divergence

## Abstract

Gene duplication is a key biological process in the evolutionary history of plants and an important driving force for the diversification of genomic and genetic systems. Interactions between the calcium sensor calcineurin B-like protein (CBL) and its target, CBL-interacting protein kinase (CIPK), play important roles in the plant’s response to various environmental stresses. As a food crop with important economic and research value, turnip (*Brassica rapa* var. *rapa*) has been well adapted to the environment of the Tibetan Plateau and become a traditional crop in the region. The *BrrCIPK9* gene in turnip has not been characterized. In this study, two duplicated genes, *BrrCIPK9.1* and *BrrCIPK9*.*2*, were screened from the turnip genome. Based on the phylogenetic analysis, *BrrCIPK9.1* and *BrrCIPK9*.*2* were found located in different sub-branches on the phylogenetic tree. Real-time fluorescence quantitative PCR analyses revealed their differential expression levels between the leaves and roots and in response to various stress treatments. The differences in their interactions with BrrCBLs were also revealed by yeast two-hybrid analyses. The results indicate that *BrrCIPK9.1* and *BrrCIPK9*.*2* have undergone Asparagine–alanine–phenylalanine (NAF) site divergence during turnip evolution, which has resulted in functional differences between them. Furthermore, *BrrCIPK9.1* responded to high-pH (pH 8.5) stress, while *BrrCIPK9.2* retained its ancestral function (low K^+^), thus providing further evidence of their functional divergence. These functional divergence genes facilitate turnip’s good adaptation to the extreme environment of the Tibetan Plateau. In summary, the results of this study reveal the characteristics of the duplicated *BrrCIPK9* genes and provide a basis for further functional studies of *BrrCBLs–BrrCIPKs* in turnip.

## 1. Introduction

Gene duplication accelerates the generation of duplicated genes in plants, leading to new traits and contributing to species evolution. Thus, gene duplication has important implications for plant differentiation and diversity [1]. The duplicate genes generated in plants following whole-genome duplication (WGD) events can evolve new functions through gene recombination, mutation, and dislocation, with important implications for plant divergence and diversity [1,2,3]. The evolution of duplicated genes is influenced by several factors, with differences in the expression patterns of the duplicates and changes in the characteristics of the genes/proteins themselves being the main factors leading to their functional divergence [4,5,6,7]. Previous studies on cabbage, citrus, and *Medicago* have demonstrated that duplicated genes exhibit different expression profiles under various stress treatments [8,9,10]. *Arabidopsis thaliana* has undergone at least three WGD events, according to its genomic data analyses [11,12]. During evolution, Brassica crops, in addition to the presence of polyploidization events (γ, β, α) common to the ancestor of *A. thaliana* [13], also underwent their own unique genome-wide triploidization event (WGT) 13–17 million years ago [14]. In the genome of rice (*Oryza sativa* L.), 62% of the loci are duplicated, and two major duplication events have occurred during its evolutionary history. The genome-wide doubling of rice occurred before the differentiation of grasses and after the differentiation of dicotyledonous plants, about 50–70 million years ago [15]. Similar to the differences in the transcription profiles of duplicated genes, changes in gene and protein characteristics, such as mutations at various amino acid sites [6], structural divergence between introns and exons, new regulatory elements [5], and genes with higher GC levels [4], are also signs of functional divergence. Differences in the characteristics of duplicated genes can result in their interactions with other proteins, thereby contributing to a greater degree of functional divergence and the new functions of the gene product [16]. Therefore, research on the new functionalization of duplicated genes and changes in their interacting proteins caused by altered gene or protein characteristics is important for understanding the evolutionary mechanism of gene duplication, which can also provide new information about how the divergence of new features leads to new functions.

*Brassica* crops have evolved due to specific genome-wide triplication events, which are considered the ideal model for studying genome polyploidy and duplicated genes [14,17]. In addition, *Brassica* crops are closely related to *A. thaliana*, and information about the related genes in *A. thaliana* can provide a reference for research on duplicated genes in *Brassica* crops. In addition to the ancient genome polyploidy events experienced by Cruciferae, *Brassica L* experienced an additional recent genome-wide triploidy event (WGT) about 10 million years ago [14]. In a previous study on cabbage, analysis of the calcium sensor calcineurin B-like proteins (CBLs)–CBL-interacting protein kinases (CIPKs) signaling network, which is involved in responses to stress response, revealed a large number of duplicate genes in two gene families. Specifically, 19 *BrrCBLs* and 51 *BrrCIPKs* were found in turnip [18], and 18 *BraCBLs* and 47 *BraCIPKs* were found in *Brassica* crop cabbage [8]. Duplicate genes respond differently to different stressors. For example, the regulatory network associated with low temperatures encompasses more duplicated genes at multiple important nodes in kale-type oilseed rape (*Brassica napus*) than in *Arabidopsis* [19]. Calcium ions, Ca^2+^, are the most abundant second messenger in plant systems and are involved in various physiological regulation and stress response processes during plant growth and development. The signaling system consists of CBLs and CIPKs, which serve as key regulator nodes of multiple stress signaling pathways in plants [20,21,22]. CIPKs are a class of proteins that specifically bind to CBLs. CIPKs typically consist of an N-terminal kinase domain containing a typical activation loop and a C-terminal regulatory domain [23]. CBLs interact with CIPKs by binding to their NAF domain, and this region is crucial for their interaction [24,25]. The CBL–CIPK interaction network is a key part of the plant response to different abiotic stresses. The CBL–CIPK module is capable of responding to different stresses (e.g., drought, cold, heat, salinity, and pathogens). Specifically, it participates in the response to low-temperature and salt stress in citrus [9], in the regulation of potassium ion (K^+^) transport in cotton (*Gossypium hirsutum*) [26], and in the response to drought, salt, and abscisic acid (ABA) stresses in *Medicago* [10]. The CBL–CIPK module positively regulates osmotic, salt, drought, and cold tolerance, and causes a decrease in the level of gene expression as well as a decrease in the amount of gene product (RNA or protein) in heat and fungal stress in *Arabidopsis;* meanwhile, *AcCIPK* expression affects the expression of ABA-related genes and reactive oxygen species homeostasis. *AcCIPK5* confers salt, osmotic, and cold stress tolerance in transgenic *Arabidopsis* [27]. The overexpressing and complement lines in *Arabidopsis* have revealed that *BrrCBL9.2* could improve salt tolerance in *Arabidopsis*, while *BrrCBL9.1* could not [18]. The expression profiles of CIPK and CBL are good indicators for studying the response of plants to abiotic stresses and the regulation of the response, facilitating functional studies of members of the CIPK–CBL model [28].

Turnip (*B. rapa* var. *rapa*) belongs to the genus *Brassica* in the family *Brassicaceae*. It is a biennial tuberous plant used as food and fodder as well as for medicinal purposes. Turnip is grown widely in Yunnan, Guizhou, Tibet, and other high-altitude areas of China, and also has a long history of cultivation in a few low-altitude areas such as Jiangsu and Zhejiang. As a traditional crop of the Tibetan plateau, turnip is well adapted to the extreme environment of the region. It has undergone a large number of paleopolyploidization events, leading to the occurrence of duplications, losses, mutations, and chromosomal rearrangements in the genome, which facilitate its rapid genetic evolution and substantial genetic and species diversity [29,30]. A previous study on the CBL–CIPK signaling network in turnip revealed that the two gene families contain many duplicated genes, with 19 *BrrCBLs* and 51 *BrrCIPKs* in turnip, compared with 10 *AtCBLs* and 26 *AtCIPKs* in *A. thaliana* [18]. Differences in expression patterns have been found between the duplicated genes, and their products exhibit altered BrrCBL–BrrCIPK interactions. Among them, the duplicated genes *BrrCBL9.1/BrrCBL9.2* are overexpressed in *A. thaliana*; BrrCBL9.2 is able to specifically interact with some BrrCIPKs and is expressed at a higher level than *BrrCBL9.1* in response to salt stress. Previous studies have been conducted to unravel the differences in the gene expression patterns between duplicate genes in different gene families and their potential roles in the adaptation of turnip to the extreme environment of the Qinghai–Tibetan Plateau. However, there is still a lack of in-depth research on the functional divergence of duplicate genes as a result of their altered characteristics [18].

In this study, we identified and functionally analyzed the duplicated genes of *BrrCIPK9* (*CIPK9.1* and *CIPK9.2*) in the turnip genome. We determined their sequences, their location in the chromosomes, and their phylogenetic relationships, and analyzed their transcriptional profiles in different tissues (roots and leaves) in response to various stress treatments (low K^+^, low magnesium, alkalinity, and cold temperature). To explore the functions of these two genes, we investigated the interactions between each gene product and BrrCBLs based on the yeast two-hybrid analyses, and overexpressed them in *Arabidopsis*. The results of this study reveal details of the functional divergence between the duplicated genes *BrrCIPK9.1/9.2* in turnip, and provide new information about the adaptive mechanism of turnip in the Qinghai–Tibetan Plateau and surrounding areas.

## 2. Materials and Methods

### 2.1. Identification and Structural Analyses of BrrCIPK9 Genes in Turnip

The *CIPK9* gene in *A. thaliana* was downloaded from TAIR (http://www.arabidopsis.org) and used as a query to search the turnip genome within the NCBI BLAST Software and Databases, the URL of which is ftp://ftp.ncbi.nlm.nih.gov/blast/executables/blast+/LATEST/; the local database blast software for ncbi-blast is 2.2.29+-win64.exe (64-bit WINDOWS) and the basic process is software download and installation, the configuration of the local database, the creation of a query sequence file, the running of the blast command, and the viewing of the results. The command used was Blastn query AtCIPK9.txt db Brgenomedbout-result.txt. This resulted in the identification of *BrrCIPK9.1/9.2*, both containing sequences encoding the conserved NAF structural domain. A homology analysis was conducted between *AtCIPK9* and *BrrCIPK9.1/9.2* using the DNAMAN software (LynnonBiosoft, San Ramon, CA, USA, 8.0.8.789). The physicochemical parameters of the putative BrrCIPK9.1/9.2 proteins, including their molecular weight (MW), theoretical isoelectric point (pI), grand average hydropathicity, and number of amino acids, were predicted using the ProtParam tool of ExPaSy (https://web.expasy.org/protparam/) [31].

### 2.2. Phylogenetic Analysis and Chromosomal Localization

The coding sequences (CDSs) of *CIPK9* genes from different families and species (*Brassicaceae* Burnett, *Poaceae* Barnhart, *Bromeliaceae* Juss., *Malvaceae* Juss., *Salicaceae* Mirb., *Euphorbiaceae* Juss., *Asteraceae* Bercht. and J. Presl, *Leguminosae* sp., Musaceae Juss.) were translated into amino acid sequences using the DNA Sequence Translation Online software (https://www.novopro.cn/tools/translate.html). All CIPK9 protein sequences were aligned using MAFFT (version 7.520) (MAFFT—multiple sequence alignment program (cbrc.jp)) and the phylogenetic tree was constructed using the MEGA11.0 software with the maximum likelihood (ML) method and 1000 bootstrap replicates [32]. To map the location of the BrrCIPK9 gene in the turnip genome, a chromosomal distribution map of the BrrCIPK9 sequence was generated using TBtools software [33].

### 2.3. Plant Material, Growth Conditions, and Stress Treatments

Turnip seeds sown in soil pots were supplied with 1/4-strength Hoagland’s nutrient solution (pH 5.8) and grown under controlled conditions (28 °C—day/25 °C—night cycle, 75–80% relative humidity, and 200 mmol photons m^−2^ s^−1^ light intensity). The stress treatments were as follows: Mg^2+^ deficiency (10 mM) [34], K^+^ deficiency, abscisic acid (ABA) (0.2 µM, 1 µM) [35], NaCl (100 mM) [36], alkaline conditions (pH 8.0) [37], and a cold temperature (4 °C). Each type of stress treatment was applied to 20 trays, with 10 seedlings per tray (*n* = 200). The seedlings were subjected to each stress treatment at 10 days after germination. In each stress treatment, the roots and leaves of the plants were collected at 0 h (control), 0.5 h and 1 h. Samples were collected with three replicates. All samples were immediately frozen in liquid nitrogen and then stored at −80 °C until RNA extraction.

### 2.4. RNA Extraction and Quantitative Real-Time PCR (qRT-PCR) Analysis

Total RNA was extracted using the Eastep^®^ Super Total RNA Extraction Kit (Promega, Madison, WI, USA), and then quantified using a NanoDrop2000 instrument (Nano Drop Technologies, Inc., Wilmington, DE, USA). The RNA integrity was checked using 0.8% agarose gel electrophoresis. The Go Script Reverse Transcription System (Promega) was used to reversely transcribe RNA into cDNA. The qPCR analyses were conducted using the FastStart Universal SYBR Green Master mix (Roche, Mannheim, Germany) on a 7500 Sequence Detection System (Applied Biosystems, Waltham, MA, USA). Each sample was analyzed in triplicate. The thermal cycling conditions were as follows: 95 °C for 10 min, followed by 40 cycles of 94 °C for 5 s, and 60 °C for 15 s. The gene encoding *B. rapa* tubulin β-2 chain-like (LOC103873913) was amplified as an internal control. The relative gene transcript levels were calculated using the 2^−ΔΔCT^ method and histograms were plotted using the SigmaPlot 12.0 software (https://systat-sigmaplot.com). The primers used for qPCR are listed in Table S1.

### 2.5. BrrCIPK9s-CBLs Yeast Two-Hybrid Assay

The MatchMaker Y2H system was used to perform the yeast two-hybrid assays. The CDSs of *BrrCIPK9.1/9.2* and their mutated sequences (*BrrCIPK9.1C*, *BrrCIPK9.1N*, *BrrCIPK9.2C*, *BrrCIPK9.2N*, *BrrCIPK9.2E-A*, and *BrrCIPK9.2deEF*) were first subcloned individually into the *pGBKT7* vector. The CDS regions of the *BrrCBLs* genes were previously subcloned into the *pGADT7* vector [18]. Plasmids containing *BrrCIPK9.1/9.2* and their mutated variants were transformed into the yeast strain AH109 according to the methods described in the Yeast Protocol Handbook (lithium acetate transformation; Clontech). The transformed yeast strains were inoculated into specific media for growth: (1) Medium lacking leucine or tryptophan (SD−Trp−Leu) to serve as a positive control for transformation and loading; (2) medium lacking leucine, tryptophan, or histidine (SD−Trp−Leu−His) to test for protei–protein interactions under low stringency; and (3) medium lacking leucine, tryptophan, histidine, or adenine (SD−Trp−Leu−His−Ade) to test for protein–protein interactions under stringent conditions [18]. The growth of the colonies on the various media was observed every 48 h for 6 days. The primers used for vector construction are listed in Table S1.

### 2.6. Subcellular Localization of BrrCIPK9s

According to the *BrrCIPK9* sequence, specific primers were designed to amplify the CDSs of *BrrCIPK9.1/9.2*. The CDS of each gene was transformed into *Escherichia coli DH5α*. The plasmids were extracted from the *E. coli* cultural broth and electro-transferred into *Agrobacterium tumefaciens* EHA105 to obtain *A. tumefaciens* containing *EHA105-GFP-BrCIPK9.1/9.2*, which were further introduced into *A. thaliana* using the floral-dip method. Seeds of the *BrrCIPK9*-overexpressing and wild-type (WT) *A. thaliana* lines were germinated on 1/2-strength Murashige and Skoog (MS) medium. The roots reaching 2 cm in length were cut off for subcellular localization analysis. To determine the subcellular localization in the leaves, *A. tumefaciens* containing *EHA105-GFP-BrrCIPK9.1* or *9.2* was injected into the air space on the abaxial side of *Nicotiana benthamiana* leaves. After 3–5 days, the GFP fluorescence was detected by laser scanning confocal microscopy (Olympus Optical, Tokyo, Japan). The primers used for vector construction are listed in Table S1.

### 2.7. Overexpression of BrrCIPK9 Genes in Arabidopsis

Using the *Agrobacterium* strains harboring *A. tumefaciens* containing *EHA105-GFP-BrrCIPK9.1/9.2*, transgenic *Arabidopsis* plants were obtained using the floral-dip method [38]. The seeds were collected and those harboring *A. tumefaciens* containing the *EHA105-GFP-BrrCIPK9.1/9.2* vector were identified by PCR. The wild type (WT) *Arabidopsis* and the lines overexpressing each target gene were grown on 1/2 MS solid medium for analysis of their stress responses. The root length was measured after 15 days in the control medium, under alkaline stress (pH 8.5), with a lack of magnesium, under 5 µM of ABA stress, under low-temperature stress (4 °C), or under low-K^+^ (10 mM of KCl) stress. All data are expressed as mean ± standard deviation of three biomass replicates. Statistical analyses were performed using one-way ANOVA. SPSS 22.0 statistical software (SPSS Inc., Chicago, IL, USA) was used to process the experimental data. One-way analysis of variance (ANOVA) was used to compare significant differences between all groups using Tukey’s analysis.

## 3. Results

### 3.1. Characterization and Phylogenetic Relationships of BrrCIPK9 Genes

**Figure 1:**
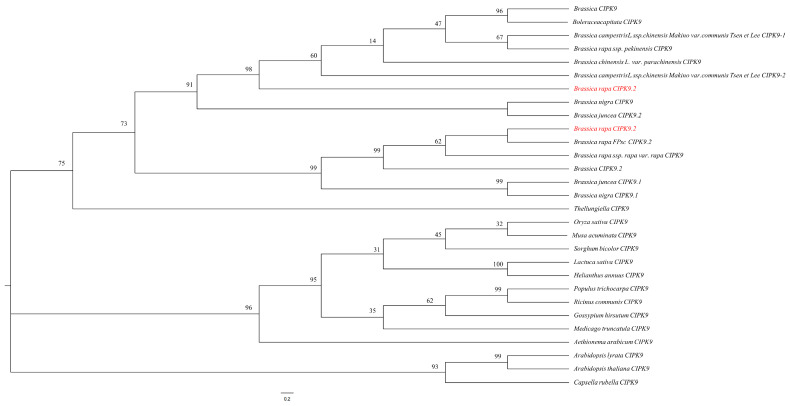
Phylogenetic relationship of CIPK proteins. The CIPK protein sequences were aligned using the MAFFT version 7 program, and phylogenetic trees were constructed using the MEGA 11.0 software with the ML method and 1000 bootstrap test replicates. The red text means our genes.

To gain insights into the phylogeny of CIPK9s, the ML method and MEGA11.0 software were used to phylogenetically analyze the CIPK9 protein of turnip and other species. The analysis of the *BrrCIPK9.1/9.2* and *AtCIPK9* collinearity results demonstrated that *BrrCIPK9s* are a set of duplicate genes (Figure S4), and that *BrrCIPK9.1* and *BrrCIPK9.2* are distributed in different branches of the tree (Figure 1). Combined with the amino acid sequence comparison results, these findings provide further evidence of the substantial differences between the two proteins.

The *Arabidopsis CIPK9* CDS sequence was used as a query in searches of the published turnip genome [34]. We identified two copies of *BrrCIPK9* (*BrrCIPK9.1* and *BrrCIPK9.2*). Both genes showed sequence similarities with *AtCIPK9*. The full-length BrrCIPK9.1 and BrrCIPK9.2 proteins were predicted to be 1338 and 1284 amino acids in length, respectively, with a predicted PI of 5.03 to 5.05 (Table S2).

**Figure 2:**
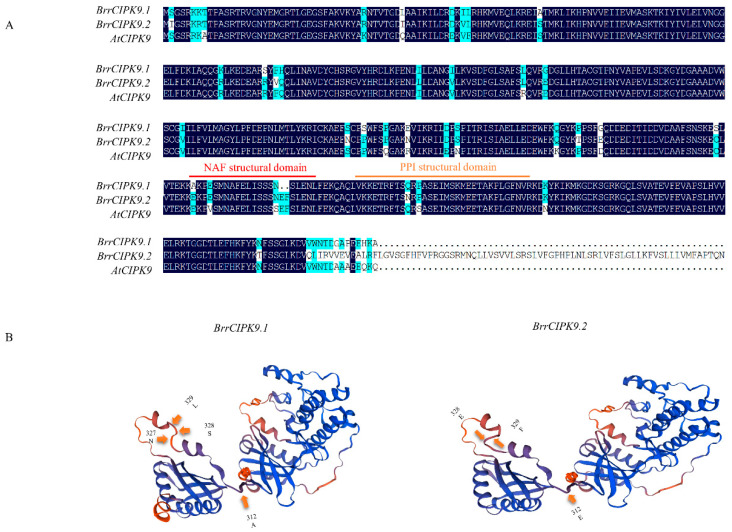
(**A**) Multiple sequence alignments of two *BrrCIPK9s* amino acids. The conserved NAF/FISL and protein–phosphatase interaction (PPI) motifs of CIPK are marked by dots above the sequence. (**B**) BrrCIPK9.1/9.2 three-dimensional construction structure prediction.

To identify the amino acid sequence differences between BrrCIPK9.1 and BrrCIPK9.2, we compared them with AtCIPK9. The similarity between AtCIPK9 and BrrCIPK9.1 was 92.39%, and that between AtCIPK9 and BrrCIPK9.2 was 80.23% (Table S2). Different colors indicate different motifs and the size of the squares indicates the motif length. Figure S1 indicates that there are differences in the motif structure between BrrCIPK9.1 and BrrCIPK9.2 and AtCIPK9. Among them, the BrrCIPK9.2 protein is more different from the AtCIPK9 protein (Figure S1). Differences in the amino acid sequence of the NAF domain were found between BrrCIPK9.1 and BrrCIPK9.2. Compared with the NAF domain in BrrCIPK9.2, two missing amino acids (glutamic acid (E) and phenylalanine (F)) and one amino acid substitution at another site (alanine in BrrCIPK9.1 and glutamic acid in BrrCIPK9.2) were found in BrrCIPK9.1 (Figure 2A,B). The NAF domain of BrrCIPK9.2 was more similar to that of AtCIPK9 than that of BrrCIPK9.1. On the basis of this finding, we hypothesized that BrrCIPK9.2 retains its original function, while BrrCIPK9.1 has a differentiated function. These results identify differences in the amino acid sequences, including those in the NAF structural domain that binds to CBL, between BrrCIPK9.1 and BrrCIPK9.2.

### 3.2. Interactions between BrrCIPK9s and BrrCBLs

**Figure 3:**
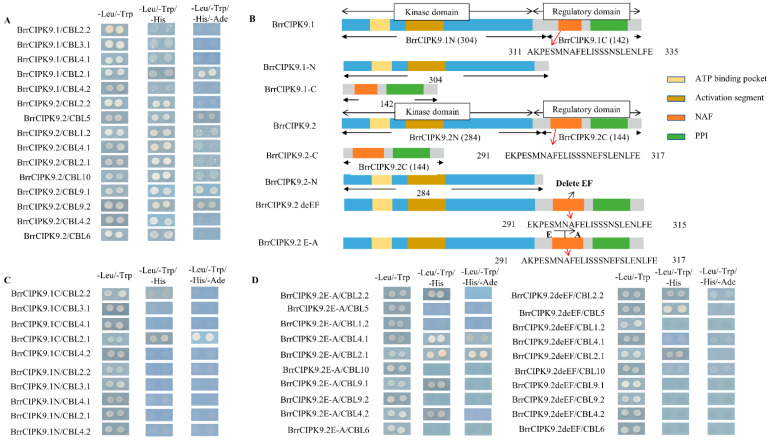
(**A**) Yeast two-hybrid results of BrrCIPK9.1 and BrrCIPK9.2 with BrrCBLs. (**B**) Structural patterns of the respective mutants of BrrCIPK9.1 and BrrCIPK9.2. (**C**) Yeast two-hybrid results of BrrCIPK9.1 mutants with BrrCBLs. (**D**) Yeast two-hybrid results of BrrCIPK9.2 mutants with BrrCBLs.

The NAF/FISL domain of the CIPK protein is involved in the interaction between CBL and CIPK. Differences between the two paralogs were identified by an amino acid sequence analysis of the NAF/FISL structural domains of BrrCIPKs. To determine whether such differences affect the interaction with BrrCBLs, we used BrrCIPK9.1, BrrCIPK9.1C, BrrCIPK9.1N, BrrCIPK9.2, BrrCIPK9.2deEF, and BrrCIPK9.2E-A with different amino acid sequences in the NAF/FISL domain for protein interaction analyses with BrrCBLs. The encoding sequence for each of them was cloned into the yeast vector pGBKT7 (Figure S2A). In total, 14 BrrCBLs were cloned and used for the Y2H assays. A comparison of the interactions between BrrCBLs and truncated and whole BrrCIPK9.1 sequences confirmed that only the sequences containing the C-terminal could interact with BrrCBLs, while the N-terminal sequences could not interact with BrrCBLs (Figure 3C). It has been shown that the NAF/FISL structural domain at the C-terminal end of CIPK proteins is the key region for the interaction between CBLs and CIPKs. Our results showed that compared with BrrCIPK9.1, BrrCIPK9.2 could interact with BrrCBLs more strongly. The differences between the NAF structural domains of BrrCIPK9.1 and BrrCIPK9.2 were further investigated by point mutation analyses. BrrCIPK9.2deEF and BrrCIPK9.2E-A were generated as mutated sequences and their interactions with BrrCBLs were determined (Figure 3D). Compared with the original sequences, those with point mutations in the NAF sequences did not or only weakly interacted with BrrCBLs. These results indicate that the three mutated loci play important roles in the interaction between BrrCIPK9s and BrrCBLs.

### 3.3. Transcriptional Profiles of BrrCIPK9 Genes in Turnip under Stress Treatments

**Figure 4:**
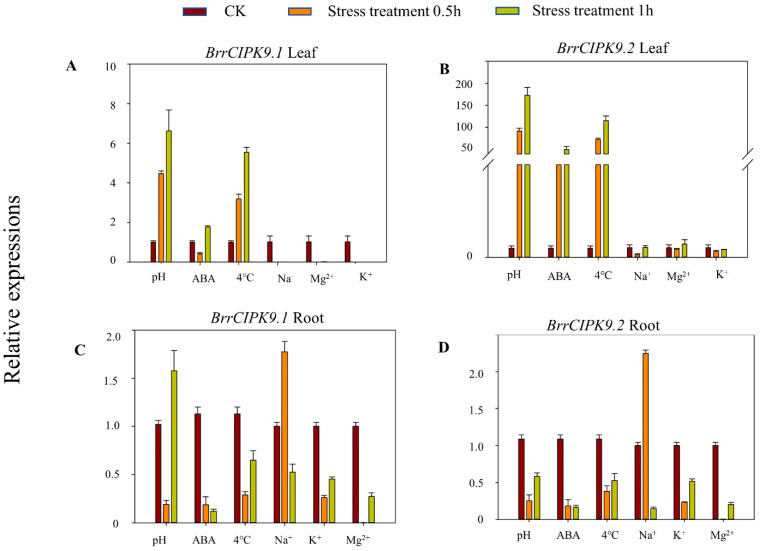
Expression profiles of *BrrCIPK9.1/9.2* genes in different stress treatments. (**A**,**B**) Expression analysis of *BrrCIPK9.1/9.2* in six different treatments at 0.5 h and 1 h in turnip leaf. (**C**,**D**) Expression analysis of *BrrCIPK9.1/9.2* in six different treatments at 0.5 h and 1 h in turnip root.

The transcriptional profiles of the *BrrCIPK9* genes in the roots and leaves of turnip plants under different stress conditions were investigated through qRT-PCR. In the roots, both *BrrCIPK9* genes were highly expressed under all stress treatments, and the transcript level of *BrrCIPK9.1* was higher than that of *BrrCIPK9.2* under alkaline stress. In the leaves, *BrrCIPK9.1* was significantly down-regulated under NaCl stress, low-MgCl_2_, and low-KCl stress, while *BrrCIPK9.1* was slightly up-regulated under alkaline, ABA, and 4 °C stress. Overall, the transcript levels of *BrrCIPK9.2* were higher than those of *BrrCIPK9.1* under different stress treatments, especially with the alkaline, ABA, and 4 °C treatments. These findings demonstrate the functional divergence between BrrCIPK9.1 and BrrCIPK9.2 (Figure 4).

### 3.4. Subcellular Localization Analysis of BrrCIPK9s

**Figure 5:**
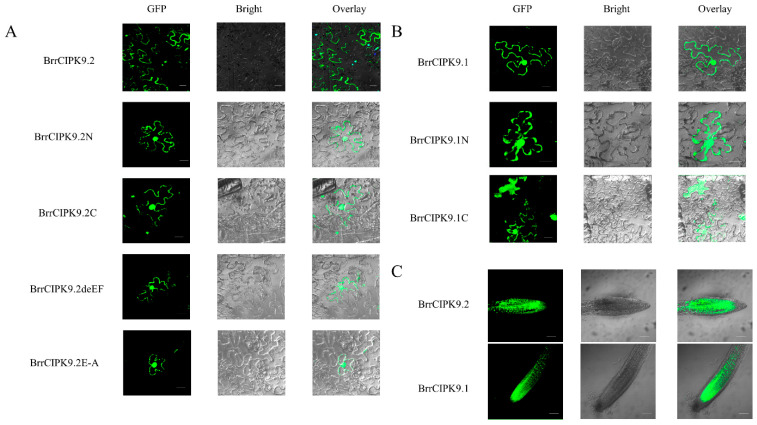
(**A**) Analysis of subcellular localization of *BrrCIPK9.2* and its mutants in *Nicotiana benthamiana* leaf cells. Bar = 20 µm. (**B**) Analysis of subcellular localization of *BrrCIPK9.1* and its mutants in *N. benthamiana* leaf cells. Bar = 20 µm. (**C**) Subcellular identification of root tip of BrrCIPK9.1/9.2 transgenic plants. Bar = 80 µm.

The subcellular localization of gene products is usually closely related to their physiological functions. The Plant-mPLoc server (sjtu.edu.cn) was used to predict the subcellular localization of BrrCIPK9.1 and BrrCIPK9.2 and their mutants. BrrCIPK9.1C was predicted to localize in chloroplasts, the cytoplasm, and the nucleus, while BrrCIPK9.1/BrrCIPK9.2 and the other mutants were predicted to localize in the nucleus. The cellular localization of BrrCIPK9 proteins was observed by laser confocal microscopy. A pRI101:BrrCIPK9-GFP vector was constructed and injected into the *N. benthamiana* leaves (Figure S2B). Transient expression in the lower epidermis of the *N. benthamiana* leaves was observed by laser confocal microscopy on day 3 after infiltration. The results showed that the BrrCIPK9.1/BrrCIPK9.2 fusion proteins emitted green fluorescent signals in the cell membrane and the nucleus of the *N. benthamiana* epidermal cells (Figure 5). These results confirm that each gene was successfully expressed in *N. benthamiana* and reveal the localization of their encoded products.

### 3.5. Functional Analysis of BrrCIPK9s under Different Abiotic Stresses

**Figure 6:**
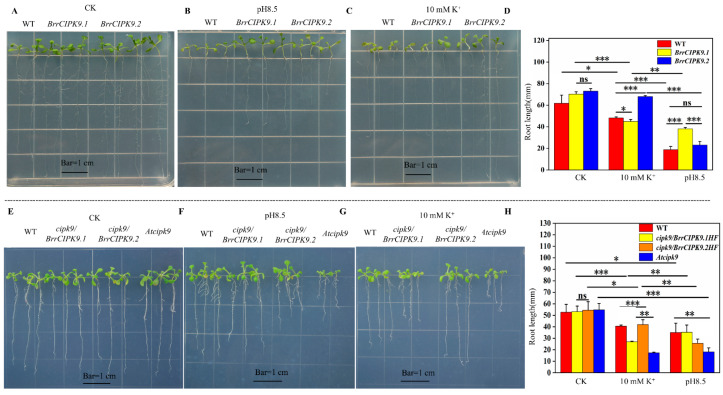
Determination of root length of t*rans-BrrCIPK9.1/9.2* and *cipk9/BrrCIPK9.1/9.2 Arabidopsis thaliana*. (**A**–**C**) Comparison of phenotypes of transgenic *BrrCIPK9.1/9.2 A. thaliana* with wild-type *A. thaliana* on 10 mM of K^+^, pH 8.5 and 1/2 MS media. Bar = 1 cm. (**D**) Comparison of root lengths of transgenic *BrrCIPK9.1/9.2* with wild-type *A. thaliana* on 10 mM of K^+^, pH 8.5 and 1/2 MS media. (**E**–**G**) Comparison of phenotypes of restoration mutation *BrrCIPK9.1/9.2 A. thaliana* with WT and *Atcipk9* on 10 mM of K^+^, pH 8.5 and 1/2 MS media. Bar = 1 cm. (**H**) Comparison of root lengths of restoration mutation *BrrCIPK9.1/9.2 A. thaliana* with WT and *Atcipk9 A. thaliana* on 10 mM of K^+^, pH 8.5 and normal MS media. WT:wild type. Asterisks indicate significant differences between treatments, with a greater number of asterisks indicating more significant differences and ns indicating no significant differences.(* *p* < 0.05 ** *p* < 0.01 *** *p* < 0.001).

To further elucidate the functional divergence between the duplicate genes, *BrrCIPK9.1* and *BrrCIPK9.2* were transformed into *A. thaliana* and the phenotypes were compared between the transgenic plants and the WT after 15 days of different abiotic stress treatments. The treatments were Mg^2+^ deficiency, K^+^ deficiency (10 mM K^+^), and alkaline stress (pH 8.5). The transgenic *BrrCIPK9.1* and *BrrCIPK9.2 A. thaliana* lines grew similarly to the WT on normal MS medium, with similar root lengths. The root length of the *BrrCIPK9.2*-overexpressing plants was greater than that of the *BrrCIPK9.1*-overexpressing plants under the Mg^2+^ deficiency, K^+^ deficiency, 100 µM of K^+^, and 10 mM of K^+^ stress treatments. In contrast, the root length of the *BrrCIPK9.1*-overexpressing plants was greater than that of the *BrrCIPK9.2*-overexpressing plants under alkaline stress (pH 8.5) (Figure 6 and Figure S5). However, there was no significant difference between the *BrrCIPK9.1*-overexpressing plants and transgenic *BrrCIPK9.2*-overexpressing plants under Mg^2+^ (10 mM), NaCl (100 mM), and ABA (0.2 µM, 1 µM) deficiency stress. Seeds from the WT, *cipk9* mutant, and restoration mutation plants were germinated on 1/2 MS culture medium, 1/2 MS culture medium with 10 mM of K^+^, and 1/2 MS culture medium at pH 8.5. The root length of the *cipk9/BrrCIPK9.1* plants was greater than that of the *cipk9/BrrCIPK9.2* plants under alkaline stress (pH 8.5). The root length of the *cipk9/BrrCIPK9.2* plants was greater than that of the *cipk9/BrrCIPK9.1* plants under alkaline stress (pH 8.5) as well. The *Atcipk9* mutant had a weak growth capacity under both abiotic stresses (Figure 6 and Figure S6). The results show that the duplicate genes *CIPK9.1* and *CIPK9.2* have undergone functional divergence.

## 4. Discussion

The high conservation of ancient duplication events and extant paired duplicated genes has contributed to the large number of duplicated genes in plant genomes. Recent WGD events have occurred in several domesticated crop species, including wheat (*Triticum aestivum*), cotton, and soybean (*Glycine max*), and have contributed to important agronomic traits such as the grain quality, fruit shape, and flowering time. The genus *Brassica* has undergone several WGD events and is therefore considered a typical species for research on duplicated genes [29,30]. In our study, we identified *BrrCIPK9.1/9.2* as a duplicated gene pair in *Brassica napus*. Duplicated genes have facilitated the evolution of novel functions, such as the production of floral structures, the induction of disease resistance, and adaptation to stress. Duplicate genes are usually expressed differently under various stress conditions. In our study, the transcript profiles of *BrrCIPK9.1* and *BrrCIPK9.2* differed between roots and leaves under different stress treatments, and their transcript levels were either increased or decreased under these tested stress treatments. The transcript levels of *BrrCIPK9.1* were very low in leaf tissues under NaCl, MgCl_2_, and KCl stress, whereas those of *BrrCIPK9.2* were relatively high. In rice, tandem repeat pairs (*OsCIPK12/30*) and fragment repeat pairs (*OsCIPK6/27*, *OsCIPK1/17*, and *OsCIPK3/31*) also show differences in their transcriptional profiles [39]. In the present study, both *BrrCIPK9.1* and *BrrCIPK9.2* were up-regulated to different extents in the roots under NaCl treatment for 0.5 h. Both *BrrCIPK9.1* and *BrrCIPK9.2* were up-regulated to different extents in the leaves under low-temperature stress. The differences in the transcriptional profiles of the duplicated genes reflect their possible functional differentiation during the adaptation of turnip to extreme environments on the Tibetan Plateau, whereas similarities in expression reflect the existence of a common response to stress.

Differences in the loci of the duplicated genes can lead to functional differences, mainly due to differences in the proteins that interact with them. Differences between duplicated genes can lead to changes in their interactions with upstream target genes. The NAF structural domain at the C-terminus of CIPK serves as a binding site for CBL, and the two gene families interact to participate in responses to different stresses [18]. In our study, we found that the C-terminus of BrrCIPK9.1 was able to interact with BrrCBLs, whereas the N-terminus was not. Structural differences are universal, and differences in structural sites lead to differences in protein interactions and thus differences in function compared with ancestral genes [40,41]. We detected site differences in the NAF structural domain between BrrCIPK9.1/9.2 and *Arabidopsis* CIPK9, as well as differences between the NAF domains of BrrCIPK9.1 and 9.2 that resulted in different interactions between BrrCIPK9.1/9.2 and BrrCBLs. These different interactions allow these genes to respond to different stresses. Duplicated genes play an important role in maintaining the stability of the plant’s genetic system and in reducing the adverse effects of the external environment on the genetic system. When one of the duplicated genes loses its original function due to mutation, the other duplicated gene retains its ancestral function and fills the gap created by the inactivation of its duplicate [42,43]. In existing studies, the C-terminus of protein kinase CIPK is a key structural domain for CBL and CIPK interactions. We verified that the NAF structural domain at the C-terminus of CIPK is the key structural domain for CBL and CIPK interactions via the segmentation of BrrCIPK9.1. The two sites mutated in BrrCIPK9.2deEF were those that differ between the BrrCIPK9.1 and BrrCIPK9.2 NAF structural domains. In the present study, the strength of the interaction between BrrCIPK9.2 and BrrCBL2.1 was weaker than that between BrrCIPK9.2E-A and BrrCBL2.1, whereas BrrCIPK9.1 strongly interacted with BrrCBL2.1. The strength of the interactions between BrrCIPK9.2 and BrrCBL2.1/2.2 were weaker than those between BrrCIPK9.2deEF and BrrCBL2.1/2.2. The NAF structural domain of BrrCIPK9.2 was processed by point mutation, and the mutated BrrCIPK9.2E-A and BrrCIPK9.2deEF were reduced in their ability to interact with BrrCBLs, demonstrating that the two sites in the NAF structural domain of BrrCIPK9 are the key sites for the interactions between BrrCIPK9.2 and BrrCBLs. Therefore, these differences in the NAF domain between BrrCIPK9.1 and BrrCIPK9.2 may lead to functional differences and thus functional differentiation.

Functional differences in duplicated genes may play an important role in plant adaptation to adverse environments. After the emergence of duplicated genes, there is a delay before they show different expression patterns and the functional differentiation of their products. Thus, when differences in their expression patterns arise, functional differentiation does not necessarily occur immediately. As evolution progresses, the duplicated genes will undergo functional differentiation, and different duplicated genes will take on different biological functions from each other. The functional divergence of duplicated genes reflects the different ways in which plant species adapt to environmental changes. The functional differences between different copies can provide plants with a greater ability to adapt to different environmental stresses. In *A. thaliana*, AtCIPK9 regulates K^+^ homeostasis in a K^+^-deficient environment [44]. VvCIPK9 from grape can improve salt tolerance in transgenic tobacco, and BnaCIPK9 regulates oil metabolism in oilseed rape [45]. We found that, compared with WT, BrrCIPK9.1-overexpressing plants showed a greater root length under alkaline (pH 8.5) stress, indicating that they were better adapted to this stressor. The BrrCIPK9.2-overexpressing plants were better adapted to the low-K^+^ environment, suggesting that BrrCIPK9.2 has similar functions to AtCIPK9. In this study, BrrCIPK9.1 and BrrCIPK9.2 responded to different stresses, with BrrCIPK9.2 retaining its ancestral function. However, BrrCIPK9.2 may be expressed at different levels under other stresses. The expression of BrrCIPK9.2 was higher than that of BrrCIPK9.1 under alkaline conditions; however, CIPK is a protein kinase, and its function depends not only on its expression, but also on its own enzyme activity, etc. [46]. The expression of both BrrCIPK9.1 and BrrCIPK9.2 was down-regulated under low-potassium stress; however, the NAF structural domain was more similar to AtCIPK9, while its transgenic plants were more adapted to the low-potassium environment. Therefore, the functional differences between the duplicates *BrrCIPK9.1* and *BrrCIPK9.2*, which are involved in different abiotic stress responses, may play an important role in the adaptation of turnip to the extreme environment of the Tibetan Plateau.

In summary, we investigated the functional differences between the duplicated genes *BrrCIPK9.1* and *BrrCIPK9.2* in turnip. Our results provide a basis for further research on duplicated genes and functional studies of CIPKs. Further research should explore the events downstream of these duplicated genes in the CBL–CIPK9 signaling system in turnip.

## Data Availability

Our data in the attached file.

## Supplementary Materials

The following supporting information can be downloaded at: https://www.mdpi.com/article/10.3390/genes15040405/s1, Table S1. The primers used in this study. Table S2. Turnip *CIPK9s* genes identified and their characteristics. Table S3. *BrrCIPK9s* gene editing. Table S4. Root length statistics of over-expression *Arabidopsis* plants under different stresses. Table S5. Root length statistics of *Arabidopsis* recovery mutant plants and *Arabidopsis* mutants under different stresses. Figure S1. A MEME analysis of motif compositions of BrrCIPK9.1/9.2 and AtCIPK9. Figure S2. (A) Full-length CDS amplification of turnip CIPK9s mutants: BrrCIPK9.1C/N, BrrCIPK9.2C/N, BrrCIPK9.2deEF, BrrCIPK9.2E-A. (B) Carrier construction model diagram. Figure S3. Transgenic BrrCIPK9.1/9.2 gene PCR identification gel map. Figure S4. Turnip CIPK9.1/9.2 Chromosome distributions. Figure S5. Determination of root length of trans-*BrrCIPK9.1/9.2 A. thaliana*. Figure S6. Determination of root length of restoration mutation *BrrCIPK9.1/9.2 A. thaliana*, wild-type *A. thaliana* (WT) and Arabidopsis mutant *Atcipk9.* Figure S7. Subcellular identification of root tip of BrrCIPK9.1/9.2 transgenic plants. Figure S8. Empty GFP positive control.
