# Supplementary material for: Functional Differentiation of the Duplicated Gene BrrCIPK9 in Turnip (Brassica rapa var. rapa)
Source: Genes (Basel). 2024 Mar 26;15(4):405. doi: 10.3390/genes15040405 (PMC11049275; doi:10.3390/genes15040405)
Supplement: Supplementary file 1 [file genes-15-00405-s001.zip › genes-2841987-supplementary.pdf]

Functional Divergence of the Duplicated Gene *BrrCIPK9* in Turnip (*Brassica rapa* var. *rapa*)

Haotong Kang [a], Yunqiang Yang [bc], Ying Meng [a*]

[a] Key Laboratory of Plant Resources Conservation and Utilization, College of Biological Resources and Environmental Sciences, Jishou University, Jishou, China
[b] The Germplasm Bank of Wild Species, Kunming Institute of Botany, Chinese Academy of Sciences, Kunming 650201, China
[c] Institute of Tibetan Plateau Research at Kunming, Kunming Institute of Botany, Chinese Academy of Sciences, Kunming, China

Corresponding author.
E-mail addresses: mengying@jsu.edu.cn (Y. Meng)

Supplemental Figures

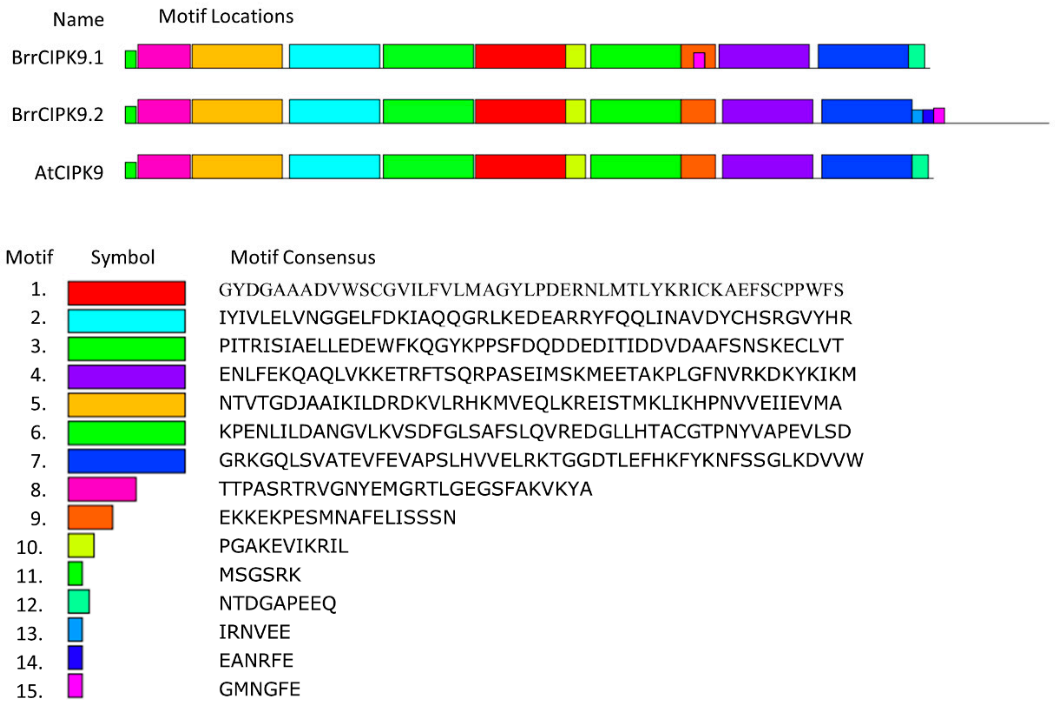

Figure S1 A MEME analysis of motif compositions of BrrCIPK9.1/9.2 and AtCIPK9.

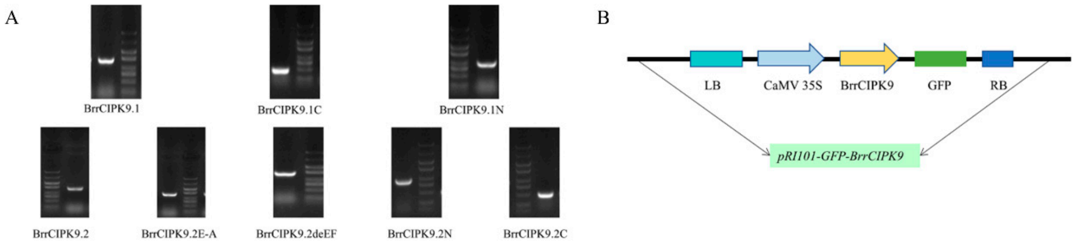

Figure S2 (A) Full-length CDS amplification of turnip *CIPK9s* mutants: BrrCIPK9.1C/N, BrrCIPK9.2C/N, BrrCIPK9.2deEF, BrrCIPK9.2E-A. (B) Carrier construction model diagram.

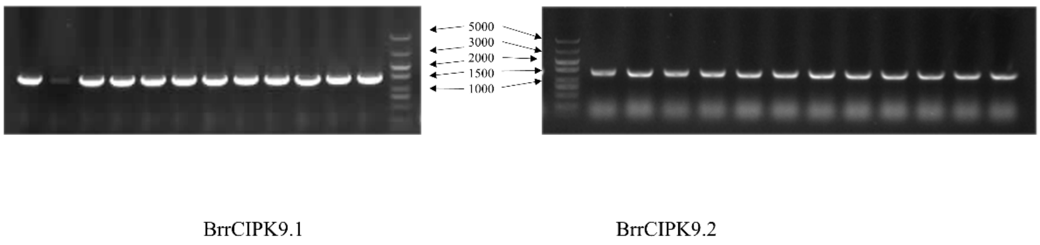

Figure S3 Transgenic BrrCIPK9.1/9.2 gene PCR identification gel map.

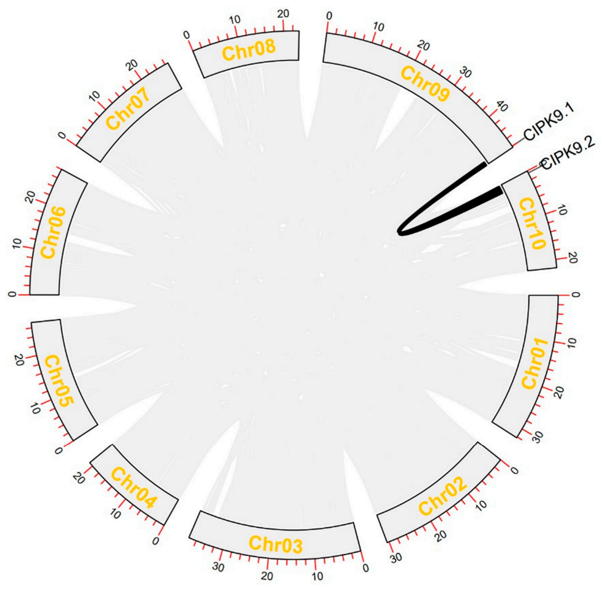

Figure S4 Turnip CIPK9.1/9.2 Chromosome distributions.

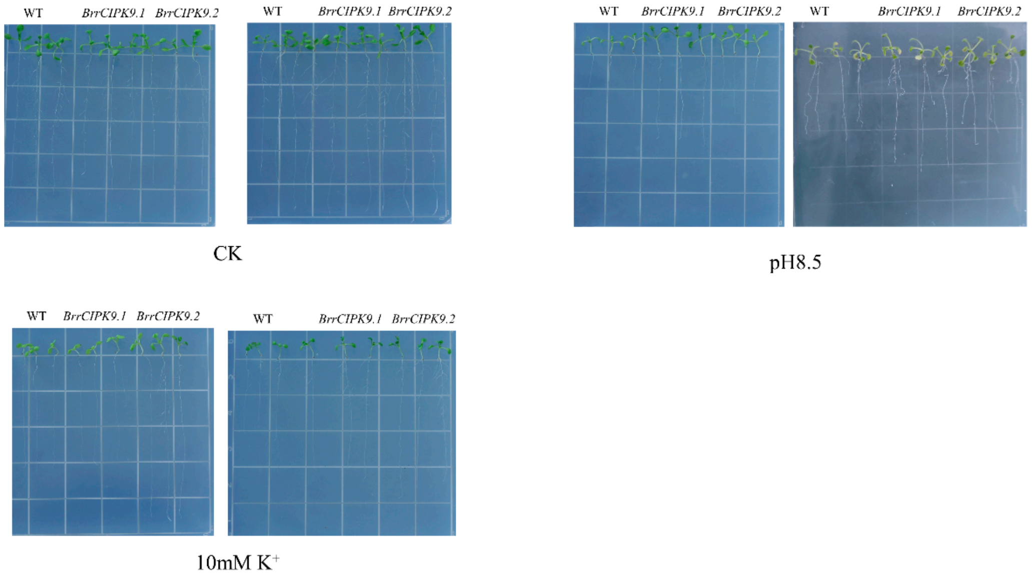

Figure S5 Determination of root length of trans-*BrrCIPK9.1/9.2* A. thaliana. We performed three repetitions, and there is a graph in the body of the text.

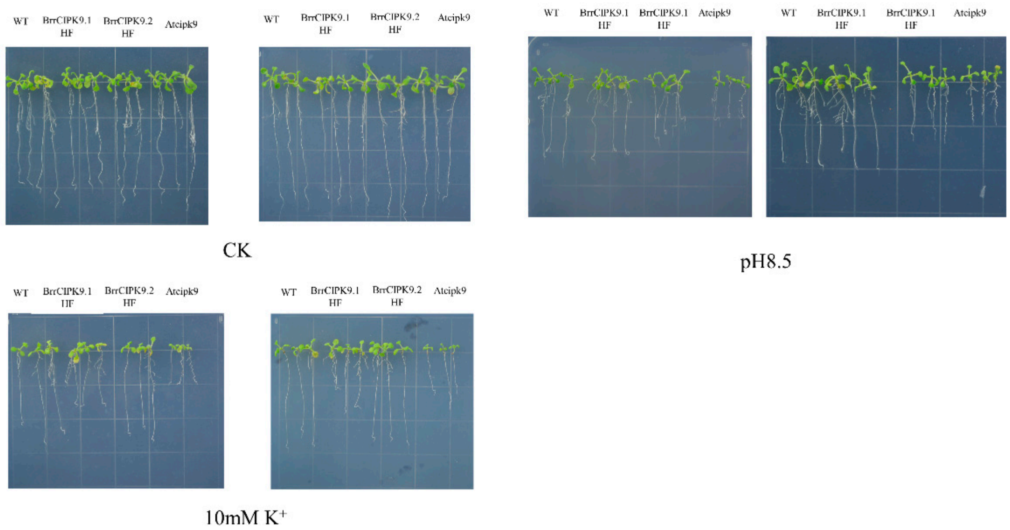

Figure S6 Determination of root length of *cipk9/BrrCIPK9.1/9.2 A. thaliana*, wild-type *A. thaliana* (WT) and Arabidopsis mutant-*Atcipk9*. We performed three repetitions, and there is a graph in the body of the text.

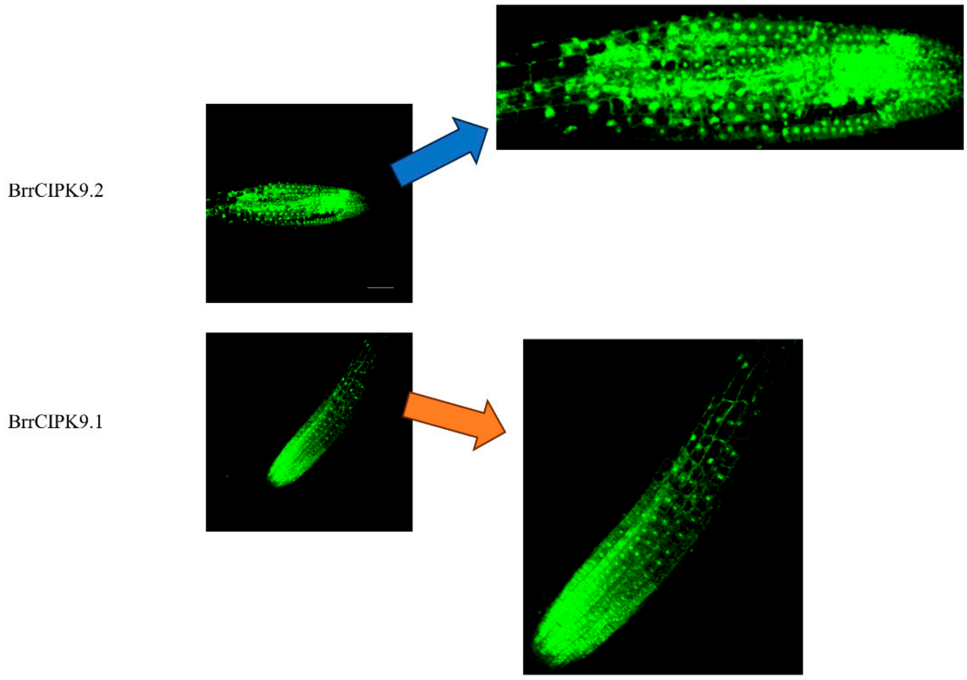

Figure S7 Subcellular identification of root tip of BrrCIPK9.1/9.2 transgenic plants.

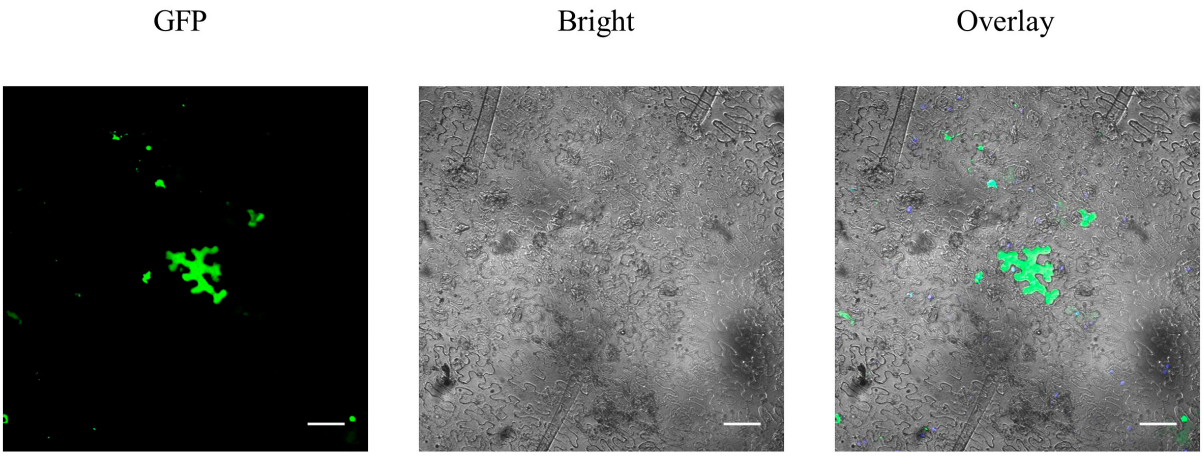

Figure S8 Empty GFP positive control.

Supplemental Tables

Table S1 The primers used in this study

Gene Name	R-terminus	F-terminus
BrrCIPK9.1-GFP	5'-GTTGATTCAGAATTCTTAAGCTTTATGTTCTTCAGG-3'	5'GCAGCGGCCGTCGACATGAGCGGGAGTAGAAAGAAG-3'
BrrCIPK9.1N-GFP	5'-GTTGATTCAGAATTCTTACTCCTTGGAGTTACTGAAAG-3'	5'GCAGCGGCCGTCGACATGAGCGGGAGTAGAAAGAAG-3'
BrrCIPK9.1C-GFP	5'-GTTGATTCAGAATTCTTAAGCTTTATGTTCTTCAGG-3'	5'-GCAGCGGCCGTCGACATGTCTCTTGTAACAGAGAAG-3'
BrrCIPK9.1-pGBKT7	5'CAGGTCGACGGATCCTTAAGCTTTATGTTCTTCAGG-3'	5'GAGGACCTGCATATGATGAGCGGGAGTAGAAAGAAG-3'
BrrCIPK9.1N-pGBKT7	5'-CAGGTCGACGGATCCTTACTCCTTGGAGTTACTGAA-3'	5'GAGGACCTGCATATGATGAGCGGGAGTAGAAAGAAG-3'
BrrCIPK9.1C-pGBKT7	5'-CAGGTCGACGGATCCTTAAGCTTTATGTTCTTCAGG-3'	5'GAGGACCTGCATATGATGTCTCTTGTAACAGAGAAG-3'
BrrCIPK9.2-GFP	5'-GTTGATTCAGAATTCTTAAACTTTATGTTCTTGAGC-3'	5'GCAGCGGCCGTCGACATGGGACGAACTCTAGGCGAAG-3'
BrrCIPK9.2N-GFP	5'-GTTGATTCAGAATTCTTATTCCTTGGAGTTACTGAAAG-3'	5'GCAGCGGCCGTCGACATGGGACGAACTCTAGGCGAAG-3'
BrrCIPK9.2C-GFP	5'-GTTGATTCAGAATTCTTAAACTTTATGTTCTTGAGC-3'	5'GCAGCGGCCGTCGACATGTGTCTTGTAACAGAGAAG-3'
BrrCIPK9.2deEF-GFP	5'-GTTGATTCAGAATTCTTAAACTTTATGTTCTTGAGC-3'	5'GCAGCGGCCGTCGACATGGGACGAACTCTAGGCGAAG-3'
BrrCIPK9.2E-A-GFP	5'-GTTGATTCAGAATTCTTAAACTTTATGTTCTTGAGC-3'	5'GCAGCGGCCGTCGACATGGGACGAACTCTAGGCGAAG-3'
BrrCIPK9.2-pGBKT7	5'-CAGGTCGACGGATCCGCTCAAGAACATAAAGTTTAA-3'	5'GAGGACCTGCATATGATGGGACGAACTCTAGGCGAAG-3'
BrrCIPK9.2N-pGBKT7	5'-CAGGTCGACGGATCCTTATTCCTTGGAGTTACTGAAAG-3'	5'GAGGACCTGCATATGATGGGACGAACTCTAGGCGAAG-3'
BrrCIPK9.2C-pGBKT7	5'-CAGGTCGACGGATCCTTAAACTTTATGTTCTTGAGC-3'	5'GAGGACCTGCATATGATGTGTCTTGTAACAGAGAAG-3'

BrrCIPK9.2deEF-pGBKT7	5’-CAGGTCGACGGATCCGCTCAAGAACATAAAGTTTAA-3’	5’GAGGACCTGCATATGATGGGACGAACTCTAGGCGAAG-3’
BrrCIPK9.2E-A-pGBKT7	5’-CAGGTCGACGGATCCGCTCAAGAACATAAAGTTTAA-3’	5’GAGGACCTGCATATGATGGGACGAACTCTAGGCGAAG-3’
qPCR-BrrCIPK9.2	5’-TAACCAGCCATGAGCACAAACAA-3’	5’-TGGGTTAAGCGCTTTCTCACTA-3’
qPCR-BrrCIPK9.1	5’-GAAGCAAACCATCTCCCCGA-3’	5’-CGAGGGGTCTACCACAGAGA-3’
qBrrTUB2	5’-ACAGGTGAAGGAATGGACGAGATG-3’	5’-AGGCGTGTGAGTGAGCAGTT-3’

Table S2 Turnip *CIPK9s* genes identified and their characteristics

Gene name	*Arabidopsis thaliana*	Identity/%	Gene locus	MW(Da)	PI	GRAVY	NO. of amino acids
BrrCIPK9.1	*AtCIPK9*	92.39%	C01:30749979..30750596 (-strand)	108843.32	5.03	0.805	1338
BrrCIPK9.2		80.23%	C09:12578364..12579026 (-strand)	104904.47	5.05	0.757	1284

Table S3 BrrCIPK9s gene editing

Gene Name	Sequence
BrrCIPK9.1-C	ATGTCTCTTGTAACAGAGAAGAAGGCGAAACCGGAATCCATGAACGCTTTTGAACTTATCTCTAGCTCAAACAGTCTTGAA<br>AACTTGTTCGAAAAGCAAGCCCAACTTGTGAAGAAAGAGACACGGTTTACTTCTCAACGACCTGCGAGCGAAATAATGTC<br>CAAAATGGAAGAAACCGCAAAGCCATTAGGCTTCAACGTCCGTAAAGACAAGTACAAGATAAAAATGAAAGGAGACAAA<br>AGTGGTCGTAAAGGCCAGCTCTCCGTTGCTACAGAGGTGTTTGAAGTGGCGCCATCGTTGCATGTAGTAGAGCTTAGGAA<br>AACCGGCGGTGATACCCTCGAGTTTCACAAGTTCTACAAAAACTTCTCATCTGGATTAAAGGATGTAGTGTGGAATACTGA<br>TGGAGCACCTGAAGAACATAAAGCTTAA
BrrCIPK9.1-N	ATGAGCGGGAGTAGAAAGAAGACAACGCCGGCAAGCCGGACGCGAGTGGGGAACTACGAGATGGGACGAACTCTAGGC<br>GAAGGAAGCTTCGCTAAGGTCAAATACGCAAGGAACACCGTCACTGGAGATCTAGCCGCTATCAAAATCCTCGACCGAGA<br>TAAGATTCTCCGCCACAAAATGGTCGAACAGCTTAAAAGAGAAATAGCGACAATGAAACTGATTAAACATCCAAATGTGGT<br>CGAAATCATTGAGGTTATGGCGAGCAAAACTAAGATCTATATTGTTCTTGAGCTGGTCAACGGAGGTGAACTATTTGATAAA<br>ATCGCCCAACAGGGGAAACTAAAGGAGGACGAAGCTCGGAGTTATTTTCATCAGCTCATAAATGCTGTGGATTATTGCCAC<br>AGTCGAGGGGTCTACCACAGAGATCTCAAGCCTGAAAATCTTATCCTTGATGCAAATGGGATTTTAAAAGTTTCTGATTTTG<br>GGCTAAGCGCGTTTTCACTACAAGTTCGGGGAGATGGTTTGCTTCACACTGCTTGTGGAACGCCGAACTATGTTGCTCCTG<br>AGGTTCTGTCGGACAAAGGCTATGACGGTGCAGCAGCAGACGTCTGGTCCTGTGGTATTATTCTATTTGTGCTCATGGCTGG<br>TTACTTGCCTTTTGATGAGCCAAATCTCATGACATTATACAAACGTATATGCAAAGCTGAGTTCAGCTGCCCATCATGGTTCT<br>CGCCCGGTGCCAAGGAAGTCATTAAGCGTATTCTTGATCCCAGTCCTATAACCAGAATAAGTATTGCAGAATTACTAGAAGA<br>TGAATGGTTCAAGCAAGGGTACAAGCCACCATCGTTTGGCCAAGATGATGAAGACATAACCATTGATGATGTGGATGCTGC<br>TTTCAGTAACTCCAAGGAGTAA
BrrCIPK9.2-N	ATGGGACGAACTCTAGGCGAAGGTAGCTTCGCTAAGGTCAAATACGCTAGGAACACCGTCACTGGAGATATAGCCGCTATT<br>AAAATCCTCGACCGGGATAAGGTTCTACGTCACAAAATGGTTGAACAGCTTAAAAGAGAAATTTCGACAATGAAACTGAT<br>TAAACATCCAAATGTGGTCGAAATCATTGAGGTTATGGCGAGCAAAACGAAGATCTATATCGTTCTTGAGCTTGTCAATGGA<br>GGTGAACTCTTTGATAAAATCGCTCAACAAGGGAGACTTAAGGAGGATGAAGCTCGGAGATATGTTCAGCAGCTCATCAAT<br>GCCGTTGATTACTGCCACAGTCGAGGGGTCTACCACAGAGATCTCAAGCCTGAAAATCTTCTTCTTGATGCAAATGGGGTT<br>TTGAAAGTTTCTGATTTTGGGTTAAGCGCTTTCTCACTACAAGTTCGGGAAGATGGTTTGCTTCACACAGCTTGTGGAACC<br>CCCAACTATGTTGCTCCTGAGGTTTTGTCGGATAAAGGCTATGACGGTGCAGCAGCAGATGTATGGTCATGTGGTGTTATTT<br>TGTTTGTGCTCATGGCTGGTTACTTGCCCTTTGATGAGCCAAATCTCATGACATTATATAAACGTATATGCAAGGCTGAATTC<br>AACTGTCCACCATGGTTCTCGCCAGGTGCCAAGAATGTCATTAAGCGTATTCTTGATCCCAGCCCTATAACCAGAATAAGTA<br>TCGCAGAGTTGCTAGAAGATGAATGGTTCAAGCAAGGGTACAAGACGCCATCATTTGAGCAAGATGATGAAGACATAACC<br>ATTGATGATGTTGATGCTGCTTTCAGTAACTCCAAGGAATAA
BrrCIPK9.2-C	ATGTGTCTTGTAACAGAGAAGAAGGAGAAACCTGAATCGATGAATGCTTTTGAACTTATCTCTAGCTCAAACGAGTTCAGT<br>CTTGAAAACTTGTTCGAGAAGCAAGCCCAACTTGTGAAGAAAGAGACGCGGTTTACTTCAAACCGACCTGCAAGCGAAA<br>TAATGTCGAAGATGGAAGAAACCGCAAAGCCATTAGGCTTCAATGTCCGTAAAGACAAGTACAAGATAAAAATGAAAGGA<br>GACAAAAGTGGTCGTAAAGGTCAGCTCTCAGTTGCTACAGAGGTGTTTGAAGTGGCACCATCTTTGCATGTGGTAGAGCT

	TAGGAAAACCGGCGGTGATACCCTCGAGTTTCACAAGTTCTACAAAACCTTCTCATCTGGATTAAAGGATGTAGTCTGGAA<br>TACTGATGGCGCAGCTCAAGAACATAAAGTTTAA
BrrCIPK9.2E-A	ATGGGACGAACTCTAGGCGAAGGTAGCTTCGCTAAGGTCAAATACGCTAGGAACACCGTCACTGGAGATATAGCCGCTATT<br>AAAATCCTCGACCGGGATAAGGTTCTACGTCACAAAATGGTTGAACAGCTTAAAAGAGAAATTTCGACAATGAAACTGAT<br>TAAACATCCAAATGTGGTCGAAATCATTGAGGTTATGGCGAGCAAAACGAAGATCTATATCGTTCTTGAGCTTGTCAATGGA<br>GGTGAACTCTTTGATAAAATCGCTCAACAAGGGAGACTTAAGGAGGATGAAGCTCGGAGATATGTTCAGCAGCTCATCAAT<br>GCCGTTGATTACTGCCACAGTCGAGGGGTCTACCACAGAGATCTCAAGCCTGAAAATCTTCTTCTTGATGCAAATGGGGTT<br>TTGAAAGTTTCTGATTTTGGGTTAAGCGCTTTCTCACTACAAGTTCGGGAAGATGGTTTGCTTCACACAGCTTGTGGAACC<br>CCCAACTATGTTGCTCCTGAGGTTTTGTCGGATAAAGGCTATGACGGTGCAGCAGCAGATGTATGGTCATGTGGTGTTATTT<br>TGTTTGTGCTCATGGCTGGTTACTTGCCCTTTGATGAGCCAAATCTCATGACATTATATAAACGTATATGCAAGGCTGAATTC<br>AACTGTCCACCATGGTTCTCGCCAGGTGCCAAGAATGTCATTAAGCGTATTCTTGATCCCAGCCCTATAACCAGAATAAGTA<br>TCGCAGAGTTGCTAGAAGATGAATGGTTCAAGCAAGGGTACAAGACGCCATCATTTGAGCAAGATGATGAAGACATAACC<br>ATTGATGATGTTGATGCTGCTTTCAGTAACTCCAAGGAATGTCTTGTAACAGAGAAGAAGGCGAAACCTGAATCGATGAAT<br>GCTTTTGAACTTATCTCTAGCTCAAACGAGTTCAGTCTTGAAAACTTGTTCGAGAAGCAAGCCCAACTTGTGAAGAAAGA<br>GACGCGGTTTACTTCAAACCGACCTGCAAGCGAAATAATGTCGAAGATGGAAGAAACCGCAAAGCCATTAGGCTTCAATG<br>TCCGTAAAGACAAGTACAAGATAAAAATGAAAGGAGACAAAAGTGGTCGTAAAGGTCAGCTCTCAGTTGCTACAGAGGT<br>GTTTGAAGTGGCACCATCTTTGCATGTGGTAGAGCTTAGGAAAACCGGCGGTGATACCCTCGAGTTTCACAAGTTCTACAA<br>AACCTTCTCATCTGGATTAAAGGATGTAGTCTGGAATACTGATGGCGCAGCTCAAGAACATAAAGTTTAA
BrrCIPK9.2deEF	ATGGGACGAACTCTAGGCGAAGGTAGCTTCGCTAAGGTCAAATACGCTAGGAACACCGTCACTGGAGATATAGCCGCTATT<br>AAAATCCTCGACCGGGATAAGGTTCTACGTCACAAAATGGTTGAACAGCTTAAAAGAGAAATTTCGACAATGAAACTGAT<br>TAAACATCCAAATGTGGTCGAAATCATTGAGGTTATGGCGAGCAAAACGAAGATCTATATCGTTCTTGAGCTTGTCAATGGA<br>GGTGAACTCTTTGATAAAATCGCTCAACAAGGGAGACTTAAGGAGGATGAAGCTCGGAGATATGTTCAGCAGCTCATCAAT<br>GCCGTTGATTACTGCCACAGTCGAGGGGTCTACCACAGAGATCTCAAGCCTGAAAATCTTCTTCTTGATGCAAATGGGGTT<br>TTGAAAGTTTCTGATTTTGGGTTAAGCGCTTTCTCACTACAAGTTCGGGAAGATGGTTTGCTTCACACAGCTTGTGGAACC<br>CCCAACTATGTTGCTCCTGAGGTTTTGTCGGATAAAGGCTATGACGGTGCAGCAGCAGATGTATGGTCATGTGGTGTTATTT<br>TGTTTGTGCTCATGGCTGGTTACTTGCCCTTTGATGAGCCAAATCTCATGACATTATATAAACGTATATGCAAGGCTGAATTC<br>AACTGTCCACCATGGTTCTCGCCAGGTGCCAAGAATGTCATTAAGCGTATTCTTGATCCCAGCCCTATAACCAGAATAAGTA<br>TCGCAGAGTTGCTAGAAGATGAATGGTTCAAGCAAGGGTACAAGACGCCATCATTTGAGCAAGATGATGAAGACATAACC<br>ATTGATGATGTTGATGCTGCTTTCAGTAACTCCAAGGAATGTCTTGTAACAGAGAAGAAGGAGAAACCTGAATCGATGAAT<br>GCTTTTGAACTTATCTCTAGCTCAAACAGTCTTGAAAACTTGTTCGAGAAGCAAGCCCAACTTGTGAAGAAAGAGACGCG<br>GTTTACTTCAAACCGACCTGCAAGCGAAATAATGTCGAAGATGGAAGAAACCGCAAAGCCATTAGGCTTCAATGTCCGTA<br>AAGACAAGTACAAGATAAAAATGAAAGGAGACAAAAGTGGTCGTAAAGGTCAGCTCTCAGTTGCTACAGAGGTGTTTGA<br>AGTGGCACCATCTTTGCATGTGGTAGAGCTTAGGAAAACCGGCGGTGATACCCTCGAGTTTCACAAGTTCTACAAAACCTT<br>CTCATCTGGATTAAAGGATGTAGTCTGGAATACTGATGGCGCAGCTCAAGAACATAAAGTTTAA

Table S4 Root length statistics of over-expression *Arabidopsis* plants under different stresses.

Conditions of treatment	*Arabidopsis thaliana* plants	Lengths (mm)		
CK	WT	59.73	57.52	60.80
	BrrCIPK9.1	54.33	57.26	55.04
	BrrCIPK9.2	56.03	60.17	54.54
pH8.5	WT	48.07	49.27	47.39
	BrrCIPK9.1	43.27	46.81	44.94
	BrrCIPK9.2	67.14	67.90	68.98
10 mM K^+	WT	15.45	20	21.01
	BrrCIPK9.1	37.87	37.00	39.49
	BrrCIPK9.2	22.10	26.85	23.08

Table S5 Root length statistics of *Arabidopsis* recovery mutant plants and *Arabidopsis* mutants under different stresses.

Conditions of treatment	*Arabidopsis thaliana* plants	Lengths (mm)		
CK	WT	45.36	58.98	53.60
	cipk9/BrrCIPK9.1	48.56	58.31	52.50
	cipk9/BrrCIPK9.2	47.46	61.94	53.98
	cipk9	49.38	60.24	54.98
pH8.5	WT	42.63	26.33	35.98
	cipk9/BrrCIPK9.1	41.28	28.81	35.70
	cipk9/BrrCIPK9.2	23.73	23.26	29.84
	cipk9	17.88	14.84	21.78
10 mM K^+	WT	39.85	40.17	41.69
	cipk9/BrrCIPK9.1	26.91	26.50	27.50
	cipk9/BrrCIPK9.2	38.44	46.61	40.63
	cipk9	17.25	16.81	17.99
